# The binding affinity of PTPN13’s tandem PDZ2/3 domain is allosterically modulated

**DOI:** 10.1186/s12860-019-0203-6

**Published:** 2019-07-08

**Authors:** Markus Dicks, Gerd Kock, Bastian Kohl, Xueyin Zhong, Stefanie Pütz, Rolf Heumann, Kai S. Erdmann, Raphael Stoll

**Affiliations:** 1 Biomolecular NMR, Faculty of Chemistry and Biochemistry, Ruhr-University of Bochum, 44780 Bochum, Germany; 2 Biochemistry II, Faculty of Chemistry and Biochemistry, Ruhr-University of Bochum, 44780 Bochum, Germany; 3 Department of Biomedical Science, University of Sheffield, S10 2TN, Sheffield, UK

**Keywords:** PTPN13, APC, PDZ2/3 tandem domain, NMR, Allosteric affinity modulation

## Abstract

**Background:**

Protein tyrosine phosphatase PTPN13, also known as PTP-BL in mice, is a large multi-domain non-transmembrane scaffolding protein with a molecular mass of 270 kDa. It is involved in the regulation of several cellular processes such as cytokinesis and actin-cytoskeletal rearrangement. The modular structure of PTPN13 consists of an N-terminal KIND domain, a FERM domain, and five PDZ domains, followed by a C-terminal protein tyrosine phosphatase domain. PDZ domains are among the most abundant protein modules and they play a crucial role in signal transduction of protein networks.

**Results:**

Here, we have analysed the binding characteristics of the isolated PDZ domains 2 and 3 from PTPN13 and compared them to the tandem domain PDZ2/3, which interacts with 12 C-terminal residues of the tumour suppressor protein of APC, using heteronuclear multidimensional NMR spectroscopy. Furthermore, we could show for the first time that PRK2 is a weak binding partner of PDZ2 and we demonstrate that the presence of PDZ3 alters the binding affinity of PDZ2 for APC, suggesting an allosteric effect and thereby modulating the binding characteristics of PDZ2. A HADDOCK-based molecular model of the PDZ2/3 tandem domain from PTPN13 supports these results.

**Conclusions:**

Our study of tandem PDZ2/3 in complex with APC suggests that the interaction of PDZ3 with PDZ2 induces an allosteric modulation within PDZ2 emanating from the back of the domain to the ligand binding site. Thus, the modified binding preference of PDZ2 for APC could be explained by an allosteric effect and provides further evidence for the pivotal function of PDZ2 in the PDZ123 domain triplet within PTPN13.

## Background

In biological processes, signal transduction depends on protein networks that include a vast number of highly specialised protein domains [1, 2]. The interaction of proteins can also be thought of as an energy distribution over intra domain pathways and binding surfaces that cause specific changes in the structure and function of proteins [3–5]. One of the most important protein modules in signal transduction are PDZ domains [1, 2], which are found ubiquitously in the eukaryotic genome. PDZ is an acronym for PSD95, a synapse associated protein, the septate junction protein Disc-large, and the tight junction protein Zonula occludens-1. The main function of PDZ domains is to provide a scaffold for membrane-associated protein complexes [2] by binding to C-terminal fragments of receptors, ion channels, and other integral membrane proteins. PDZ domains contain about 90 amino acids and they share a common globular fold that consists of two α-helices 1 and 2 (termed DA and DB) as well as six β-strands 1 to 6 (denoted from EA to EF), which form two anti-parallel β-sheets [6, 7]. PDZ domains bind to C-terminal peptides through a conserved binding loop, which consists of four amino acids that are part of binding groove formed by α-helix DA and β-strand EB. The positions P_0_ and P_− 2_ of the peptide ligand are crucial for binding and define different PDZ binding classes [8–11]. This nomenclature is based on the accommodation of the carboxy-terminal peptide ligand residues by the canonical PDZ binding cleft. Here, the carboxylate moiety of the last ligand residue, i.e. P_0_, is bound to GLGF motif of PDZ domains, which causes ligand amino acid side chains of P_0_ and P_− 2_ to be orientated towards the PDZ binding pocket and side chains in position P_− 1_ and P_− 3_ to point away from the PDZ binding cleft into the solvent [12]. Recently, more general concepts have been introduced, which do not (entirely) rely on specific definitions of PDZ ligand classes [13, 14].

Tyrosine-protein phosphatase non-receptor type 13 (PTPN13), which is known as protein tyrosine phosphatase PTP-Basophil/Basophil-like (PTP-BL) in mice, is the central scaffolding component of a supramolecular protein complex that exhibits numerous domain-specific interactions [1]. The PTPN13-interacting proteins can mainly be divided into three groups: known or potential regulators of the actin cytoskeleton, regulators of the actin and tubulin cytoskeleton, and regulators of gene transcription. Two of known ligand peptides of PTPN13 are derived from the tumour suppressor protein adenomatous polyposis coli (APC), a class I-type ligand [11, 15], and the cytosolic protein kinase C-related kinase-2 (PRK2), a class III-type ligand. The most important role of APC is the regulation of β-catenin, which is involved in cell adhesion processes via the transmembrane receptor cadherin [16]. APC also plays a role in the regulation of transcription through the transcription factor lymphocyte enhancer binding factor/T-cell factor (LEF/TCF) [17, 18]. PRK2 is a cytosolic serine/threonine kinase that is regulated by the monomeric G-protein Rho [19, 20]. For the tandem domain PDZ1/2 of PTPN13, also known as PTP-BL in mice, an allosteric effect on peptide binding has already been observed [21]. PTPN13 represents a large multi-domain non-transmembrane scaffolding protein with different functional properties [1]. The N-terminal part of this protein consists of a kinase non-catalytic C-lobe domain (KIND), followed by a four point one, Ezrin, Radixin, Moesin (FERM) domain. In addition, PTPN13 contains five PDZ domains and a C-terminal protein tyrosine phosphatase domain. It has previously been reported for other PDZ-containing proteins, e.g. the glutamate receptor interacting protein (GRIP) and X11/Mint**,** that the binding characteristics of single domains differ from PDZ tandem and that allosteric effects can indeed regulate the binding properties of PDZ tandem domains [10, 22, 23].

Here, we describe the binding characteristics of the second and third PDZ domain of PTPN13, both individually and as a PDZ2/3 tandem domain through heteronuclear multidimensional nuclear magnetic resonance (NMR) spectroscopy. Our results reveal that PRK2 is an additional binding partner of the PDZ2 domain. Furthermore, we have discovered an allosteric effect due to an interaction of the PDZ3 domain with the opposite surface of the canonical PDZ2 ligand binding site, which is structurally broadcasted to the binding pocket of PDZ2. The analysis of the interaction between PDZ2 and PDZ3 as well as with their respective ligands sheds light on the pivotal role of the PDZ2 domain within the PDZ123 cassette of PTPN13 on a molecular level.

## Results

### Peptide binding to PDZ domains of PTPN13

**Fig. 1:**
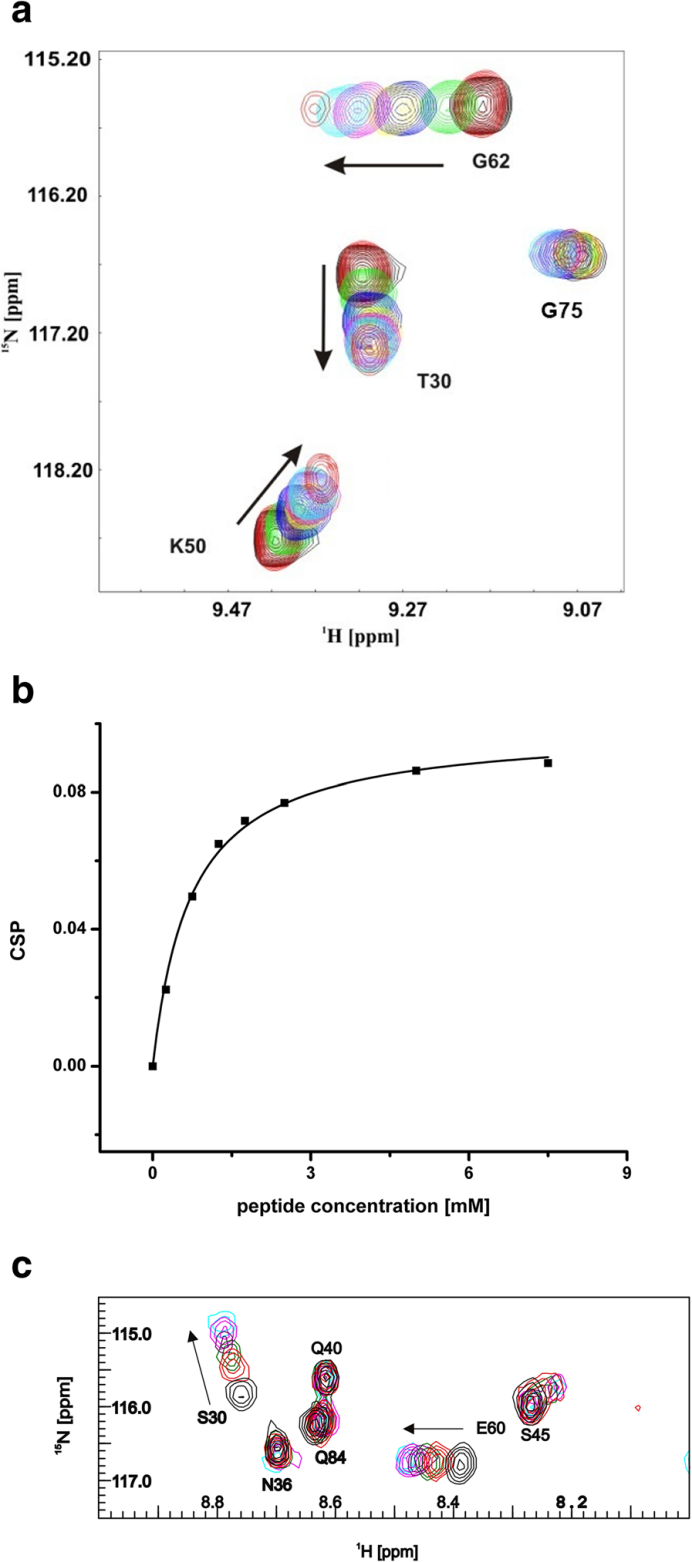
Superposition of 2D ^1^H-^15^N-HSQC NMR spectra recorded on the ^15^N-enriched single PDZ2 domain (12 kDa) recorded at 600 MHz proton frequency at 298 K and pH 7.4 upon titration with the C-terminal peptide PRK2. **a** 2D ^1^H-^15^N-HSQC NMR spectra of PDZ2 upon titration with PRK2 at various molar ratios up to 1:30 [Black (ligand free), red (1:1), green (1:3), blue (1:5), yellow (1:7), magenta (1:10), cyan (1:20), and red (1:30)]. **b** Weighted chemical shift perturbation (CSP) of residue T30 from PDZ2 as a function of PRK2 peptide concentration. Curve fitting was carried out in ORIGIN (www.originlab.com). **c** 2D ^1^H-^15^N-HSQC NMR spectra of PDZ3 upon titration with PRK2 at various molar ratios up to 1:10 recorded at 700 MHz proton frequency at 298 K and pH 7.4 [Black (ligand free), red (1:2), green (1:4), blue (1:6), magenta (1:8), and cyan (1:10)]

**Fig. 2:**
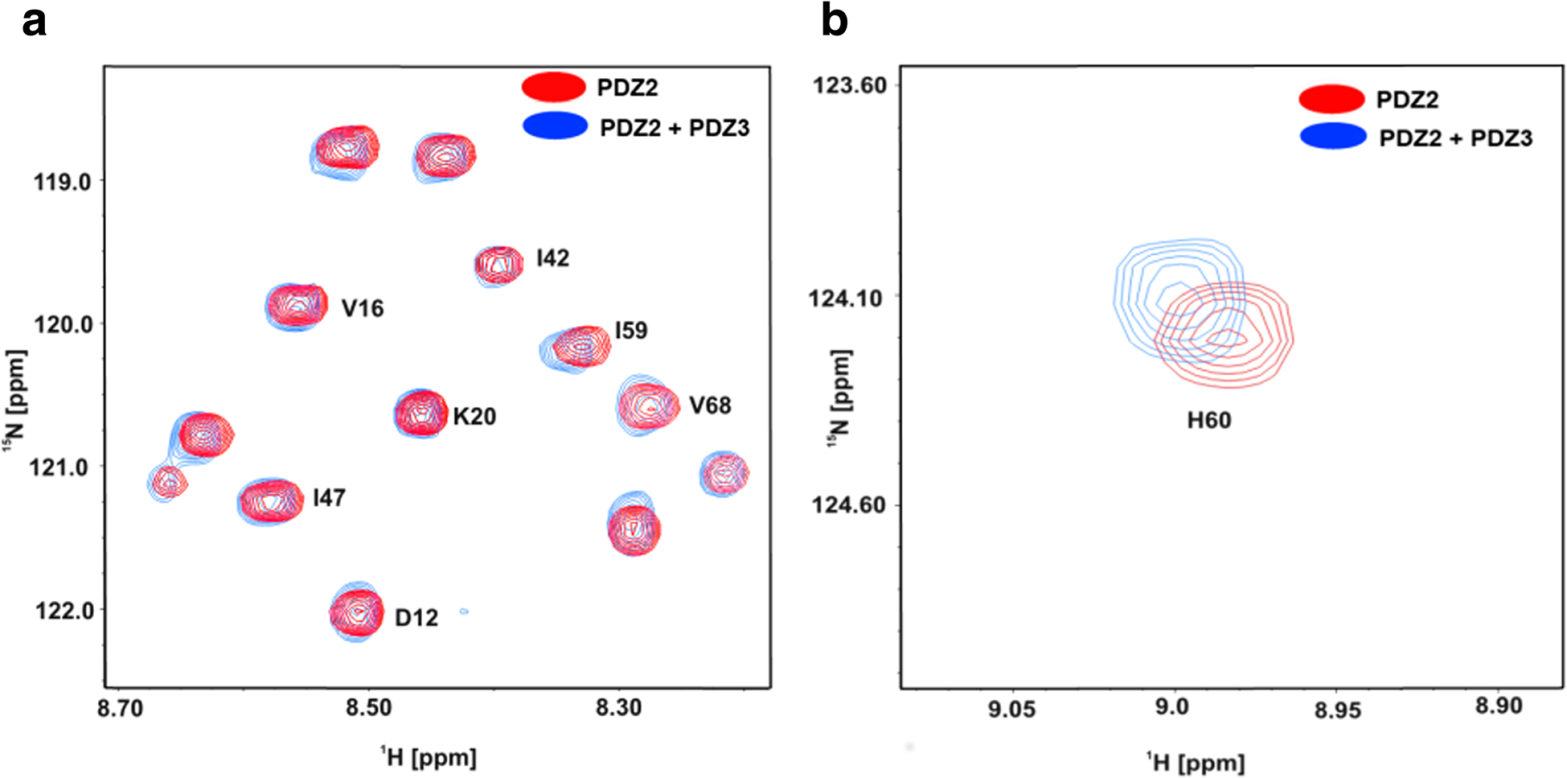
**a** Superposition of a representative region of the 2D ^1^H-^15^N-HSQC NMR spectra of PDZ2 in the absence (red) and presence of PDZ3 (blue). **b** Only very minor chemical shift differences could be observed, if at all. The final maximal stoichiometry of PDZ2 and PDZ3 was approximately 1:1

**Fig. 3:**
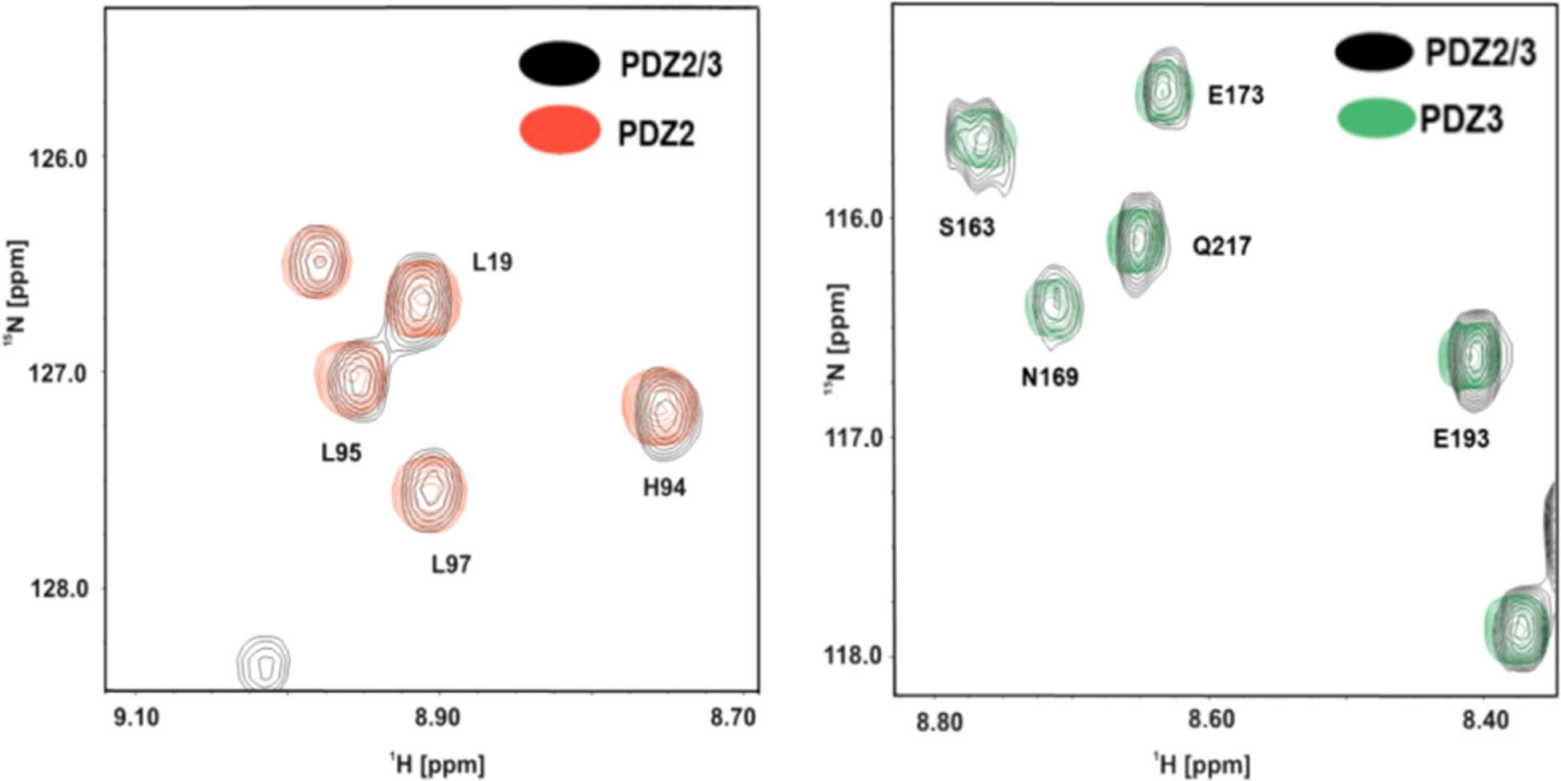
Superposition of selected regions of 2D ^1^H-^15^N-HSQC NMR spectra of PDZ2, PDZ3, and PDZ2/3. The selected regions highlight congruent NMR resonances of the apo-single domains PDZ2 (red) and PDZ3 (green) as well as the PDZ2/3 tandem domain (black)

**Fig. 4:**
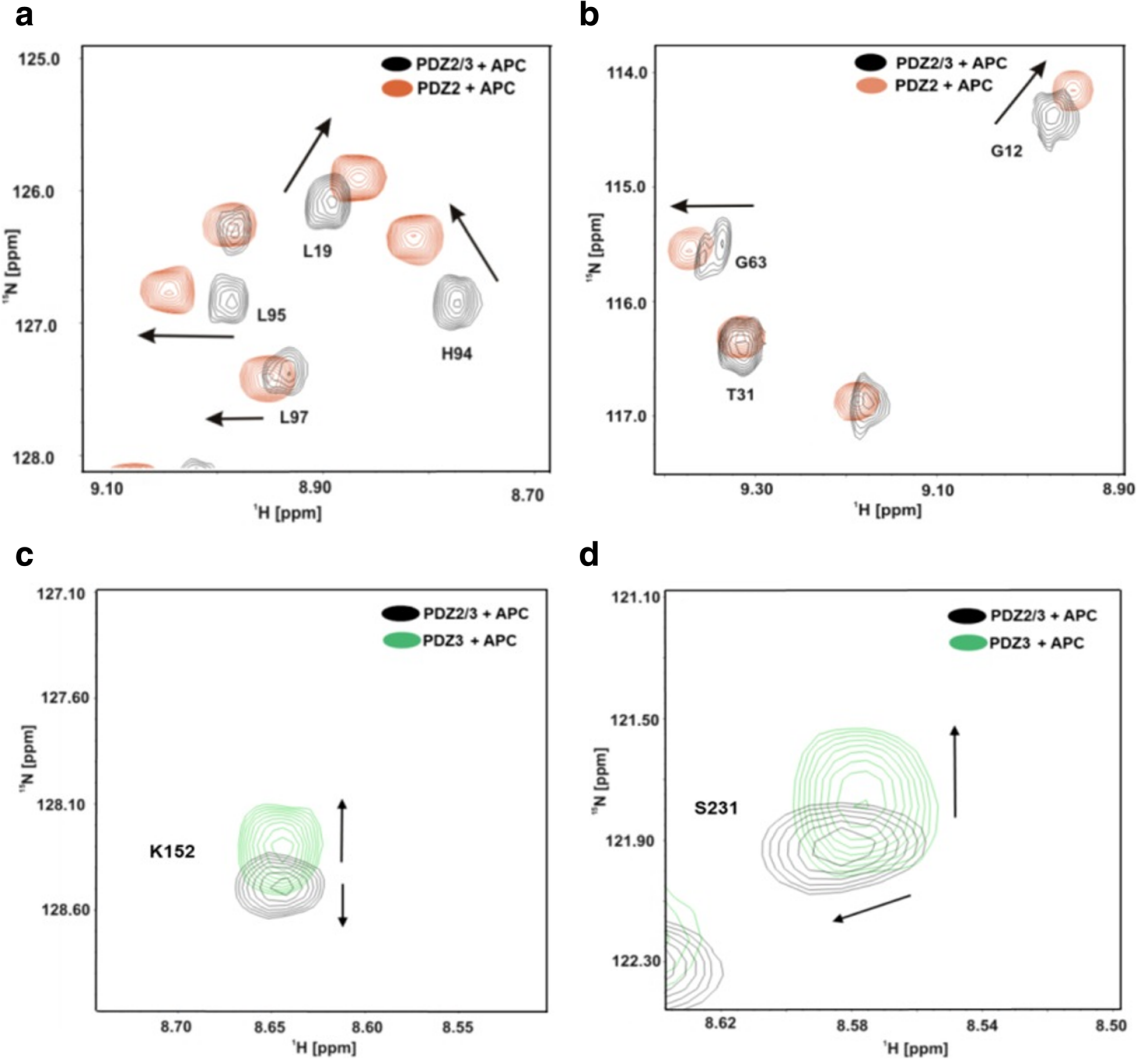
After the titration of the PDZ2 and PDZ2/3 tandem domains with the APC peptide, repectively, NMR amide backbone resonances of PDZ2 differ in their frequencies (**a**, **b**). Resonances of residues located at the back of PDZ2 domain at β-strand EA (Leu-19) and β-strand EF (His-94, Leu-95, Leu-97) are not congruent anymore. Other resonances of the PDZ2 domain, like T31, remain unaltered. NMR resonances amide backbone resonances of PDZ3 are also altered, albeit to a slightly lesser extent (**c**, **d**)

We have applied multidimensional heteronuclear NMR spectroscopy in order to elucidate the binding affinities of the PDZ2 single domain and the tandem domain PDZ2/3 of PTPN13 for the chemically synthesized C-terminal dodecapeptides derived from APC and PRK2. The binding characteristics were analyzed in a titration series of the dodecamer peptides and ^15^N isotopically-enriched PDZ2 and PDZ2/3 samples, respectively, and by monitoring the NMR chemical shift perturbations in a series of 2D ^1^H-^15^N heteronuclear single quantum coherence (HSQC) spectra with increasing ligand concentrations. It could be shown that all peptides bind to the canonical binding groove of PDZ domains (Figs. 1, 2, 3 and 4).

### Peptide binding characteristics of the PDZ2 domain

The binding study presented here revealed for the first time that the PDZ2 domain also interacts with the PRK2 peptide. The NMR chemical shift perturbations observed in 2D ^1^H-^15^N-HSQC spectra for the PDZ2-PRK2 complex demonstrate that, in comparison to PDZ3 (K_D_ = 318 ± 47 μM), the PRK2 peptide only weakly binds to the PDZ2 domain (K_D_ = 661 ± 71 μM) (Fig. 1a, b, c) [12]. In particular, NMR chemical shift differences were observed for the canonical ligand binding groove of PDZ2 including G23, S24, which are also involved in peptide recognition, and S28, and G31. Additional NMR chemical shift perturbations were observed for the β-strands EA and EF of PDZ2, located opposite to its canonical peptide binding groove. Furthermore, we titrated an APC-derived peptide, a well-known PDZ2 ligand [1], to PDZ2 and determined an affinity of 286 ± 21 μM (Table 1). Noteworthy, chemical shift changes cannot exclusively be attributed to structural changes, as structural and dynamical changes as well as direct effects of neighboring groups of the ligand all can induce chemical perturbations. Nonetheless, it is interesting to note that, upon canonical binding to PDZ2, the APC-derived peptide leads to NMR chemical shift differences observed along intra-domain signal pathways as previously predicted [3–5]. In concordance with former studies [4, 5, 24], chemical differences were observed for the binding loop and along intra-domain signaling pathways. Based on these results, dissociation constants K_D_ were extracted for the interaction of the APC-derived peptide with the PDZ2 domain (Table 1).

**Table 1:** Dissociation constants (K_D_) of APC and PRK2 for the single PDZ2 and PDZ3 [12] domains as well as for the PDZ2/3 tandem domain of PTPN13

Domain	KRHSGSYLVTSV (APC) K_D_ [μM]
PDZ2	286 ± 21
PDZ3	721 ± 148
PDZ2 in PDZ2/3	211 ± 70
PDZ3 in PDZ2/3	2574 ± 770
Domain	MFRDFDYIADWC (PRK2) K_D_ [μM]
PDZ2	661 ± 71
PDZ3	318 ± 47

### Peptide binding characteristics of the tandem domain PDZ2/3

A titration of single PDZ3 to the ^15^N isotopically-enriched single domain of PDZ2 only yielded minor chemical shift differences between the respective 2D ^1^H-^15^N-HSQC NMR spectra at a molar ratio of 1:1 (Fig. 2). Interestingly, the titration results of the tandem domain with the APC derived peptide significantly differ from that of the binding study with the isolated domain. The observed differences for APC binding to PDZ2 versus tandem PDZ2/3 are not large yet still considerable. Notably, the results listed in Table 1 indicate, that APC exhibits a slightly higher affinity to PDZ2 (K_D_ = 211 ± 70 μM) in the PDZ2/3 tandem domain than for the single PDZ2 domain (K_D_ = 286 ± 21 μM), while the extracted K_D_ value for PDZ3 (K_D_ = 2574 ± 770 μM) is even higher in comparison to the single PDZ3 domain (K_D_ = 721 ± 148 μM). Binding of APC to the tandem PDZ2/3 domain occurs in the fast exchange NMR regime. At a final stoichiometric ratio between APC and PDZ2/3 of 30:1, no further chemical shift changes were observed for amide resonances originating from the PDZ2 domain. However, due to the extremely low affinity of PDZ3 for APC, PDZ3 was not fully saturated at the end of the titration and thus represented a fractionally APC-bound state (data not shown). NMR chemical shift perturbations extracted from 2D ^1^H-^15^N-HSQC NMR spectra for the PDZ2/3 titration with the APC peptide are observed for both domains. As can be seen in Figs. 3 and 4, major chemical shift perturbations upon binding of the APC derived peptide are however mainly restricted to the PDZ2 domain. These observed chemical shifts differences of the PDZ2 domain are located at the canonical binding site and on the surface opposite to the β-strands EA and EF. Figure 4 shows representative regions of 2D ^1^H-^15^N-HSQC NMR spectra of the PDZ2/3 tandem domain upon titration with the APC peptide. For example, NMR chemical shift perturbations were observed for G12, L19, T31, G63, H94, L95, L97 of PDZ2 and K152 as well as S231 of PDZ3 in the tandem domain upon titration with APC (Fig. 4 a, b). All data are consistent with fast exchange kinetics and relatively weak (PDZ) binding affinities (Table 1).

### The interaction between PDZ2 and PDZ3 alters the binding specificity of PDZ2 in the APC-bound PDZ2/3 tandem

NMR spectroscopy was applied in order to prove as to whether the observed differences in binding affinity of PDZ2/3 for the APC derived peptide in contrast to the single PDZ2 could be explained by a PDZ domain-domain interaction. Thus, we titrated the single PDZ3 domain (residues 1491–1579) to the ^15^N-enriched PDZ2 domain (residues 1357–1442) and monitored their interaction by recording a series of 2D ^1^H-^15^N-HSQC NMR spectra (Fig. 4a). Only very minor, if at all, chemical shift differences could be observed (Fig. 4b). However, the final maximal stoichiometry of PDZ2 and PDZ3 was approx. 1:1 due to solubility issues.

**Fig. 5:**
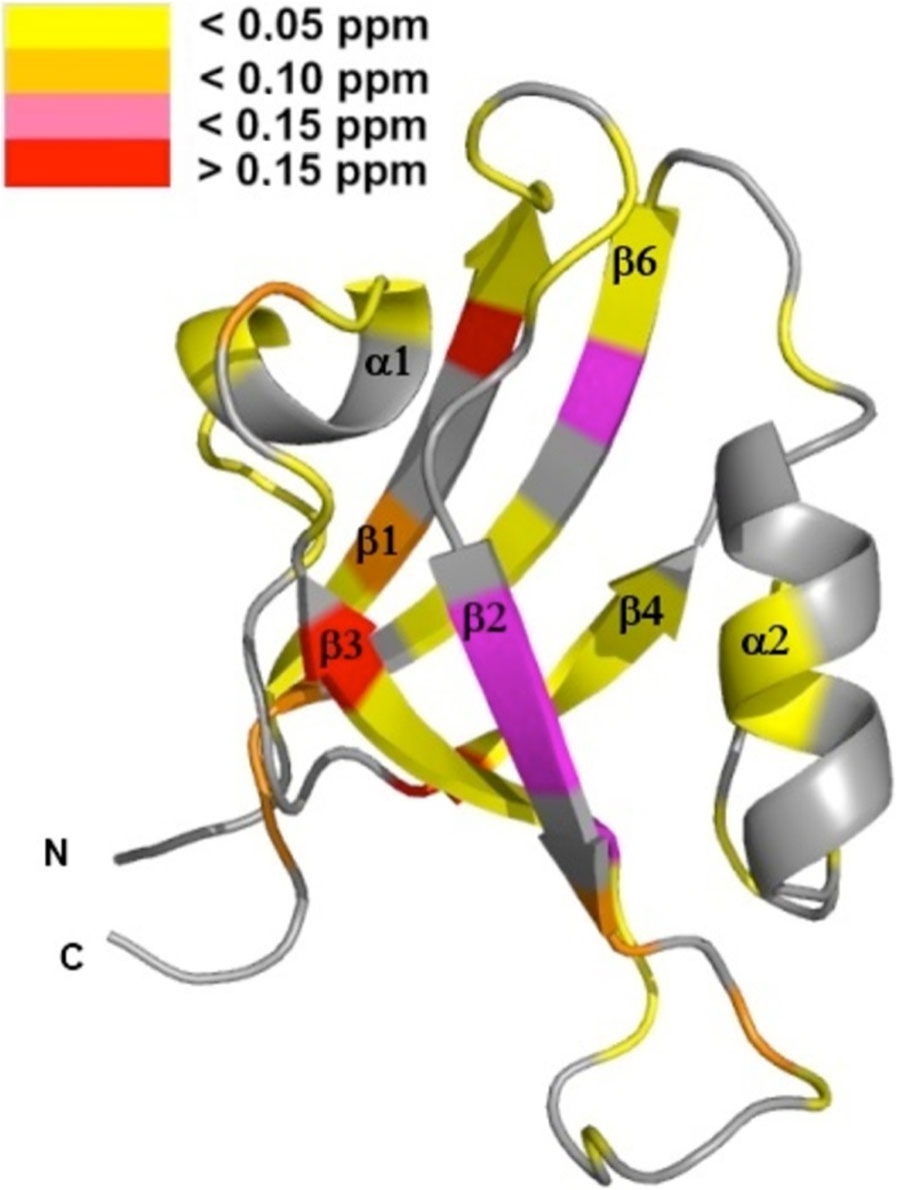
Colour-coded backbone hydrogen and nitrogen chemical shift differences between the single PDZ2-APC complex and PDZ2-APC when being part of the PDZ2/3-APC complex. The analysis is based on the structure of the single PDZ2 domain [27]. The α-helices DA and DB correspond to α1 and α2, and the β-strands EA, EB, EC, ED, EE, and EF correspond to β1 to β6, respectively. According to the given scale, the yellow to red colour gradient of the ribbon structure represents smaller to larger NMR chemical shift differences. Gray colouring indicates that either no chemical shift differences were observed or no data were available.

Furthermore, we analyzed the NMR chemical shift differences between the isolated PDZ2 domain and PDZ2 as part of the PDZ2/3 tandem domain in absence as well in the presence of the APC peptide at a molar ratio of 1:30 [25–27]. This study clearly demonstrates that the structure of the apo-PDZ2 domain does not differ from the structure of PDZ2 as part of the PDZ2/3 tandem domain, because chemical shift differences in the 2D ^1^H-^15^N-HSQC NMR spectra could not be detected (Figs. 1, 3, and 4). Obviously, a significant interaction between the PDZ domains 2 and 3 does not exist in the apo-form of the PDZ2/3 tandem domain [25]. In contrast to this, however, the protein-peptide complex of the single PDZ2 domain and the PDZ2 domain as part of the tandem domain PDZ2/3 complexed with APC peptide show NMR chemical shift differences in the respective 2D ^1^H-^15^N-HSQC spectra (Fig. 4). Major NMR chemical shift perturbations were observed on the back of the PDZ2 domain, i.e. opposite to the canonical PDZ binding cleft, mainly for EF (H94, L95, L97) and for EA (L19), while other signals do not show any chemical shift differences in the 2D ^1^H-^15^N-HSQC NMR spectra (T31) (Fig. 4 a, b). NMR resonances amide backbone resonances of PDZ3 are also altered, albeit to a slightly lesser extent (Fig. 4c, d and Fig. 5). In comparison to the apo-form of the PDZ2/3 tandem domain, the NMR line widths only slightly increase for the APC-bound form of the PDZ2/3 tandem domain (Fig. 4). This suggests an equilibrium between free and interacting PDZ domains within the PDZ2/3 tandem. Noteworthy, these NMR spectra clearly show that both PDZ domains of PDZ2/3 tandem are folded both in the free and APC-bound form (Figs. 3 and 4). Finally, the free and APC-bound NMR spectra of the individual PDZ2 domain also exclude an APC-induced dimerisation of PDZ2 (Figs. 3 and 4).

### HADDOCK-based molecular docking calculations

**Fig. 6:**
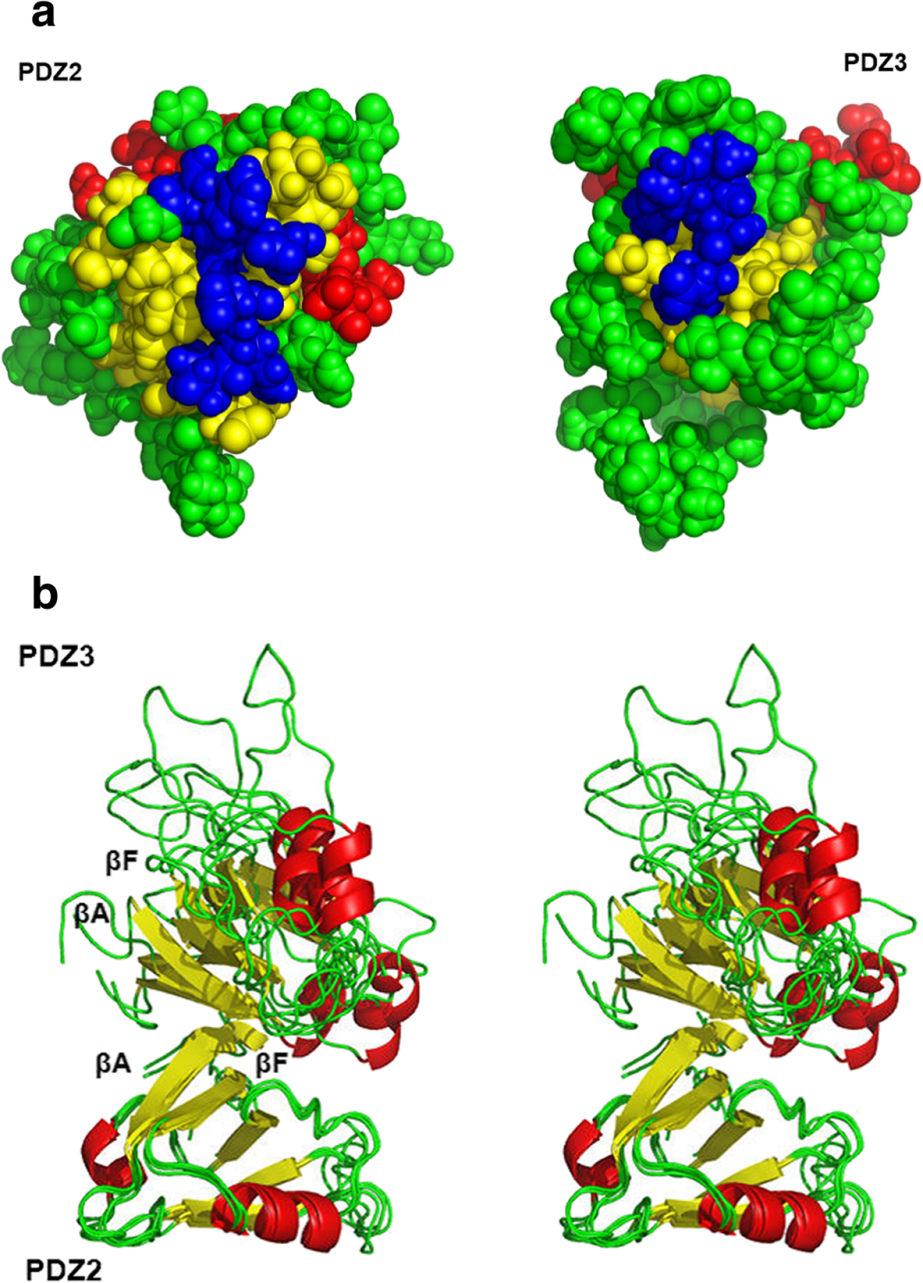
**a** For PDZ2 [27], T14, F15, E16, E18, N70, H94, and L95 were selected as AIRs in HADDOCK and are coloured in blue [28]. For a homology model of PDZ3, E17, V18, K19, L20, S98, and L100 were set as AIRs, also shown in blue. **b** HADDOCK-based [28] structural model of the PDZ2/3 tandem domain of PTPN13 in the APC-bound state. β-strands are highlighted in yellow, α-helices in red, and regions void of regular secondary structural elements in green. β-strands 1 (βA) and 6 (βF) of the PDZ2/3 tandem domain interface are indicated

A total of 10 clusters were computed, of which the best one with an overall HADDOCK software score of −92.7 ± 13.9 containing 8 individual structures was selected for further analysis (Fig. 6). The HADDOCK statistics for the APC-bound PDZ2/3 tandem domain from PTPN13 are listed in Table 2. Based on the NMR chemical shift perturbation analysis, a fully consistent molecular HADDOCK-generated model of the PDZ2/3 complex in the APC-bound state that fulfills the experimental chemical shift perturbation restraints could be calculated, in which β-strands 1 (βA) and 6 (βF) are part of the PDZ2/3 tandem domain interface (Fig. 6). The best HADDOCK cluster contains 8 structural models of the APC-bound PDZ2/3 tandem domain from PTPN13 that exhibit low van der Waals, electrostatic, desolvation, and restraint violation energies (Table 2). Noteworthy, a certain degree of rotational freedom for the two PDZ domains can be observed reflected in an RMSD value from the overall lowest-energy structure of 9.7 ± 0.7 (Table 2, Fig. 6b). Nonetheless, the canonical PDZ binding clefts are always facing the solvent (Fig. 6b). Thus, the PDZ2-PDZ3 interface is located on the opposite site with a buried surface area of 1215.5 ± 206.2 Å^2^ (Table 2).

**Table 2:** HADDOCK [28] statistics of the best cluster containing 8 structures (cluster size) of the APC-bound PDZ2/3 tandem domain from PTPN13. RMSD is the root mean square deviation of atomic positions from the overall lowest-energy structure

PTPN13 PDZ2/3 tandem domain-APC complex	
HADDOCK-Score:	−92.7 ± 13.9
Cluster Size:	8
RMSD [Å]:	9.7 ± 0.7
Van-der-Waals Energy [kcal/mol^− 1^]:	19.2 ± 13.0
Electrostatic Energy [kcal/mol^−1^]:	−463.8 ± 59.7
Desolvation Energy [kcal/mol^− 1^]:	14.5 ± 4.9
Restraint violation Energy [kcal/mol^− 1^]:	48.4 ± 42.8
Buried Surface Area [Å^2^]:	1215.5 ± 206.2

## Discussion

Several previous studies have shown that various proteins, such as PTPN13, contain multiple PDZ domainsd that these PDZ domains are clustered [1, 2]. The structures of different PDZ tandems have already been characterized to a great extent. These studies have revealed that the function of consecutive PDZ domains particularly depends on the relative orientation of the domains to one other [2, 23, 29]. For instance, the domains 4 and 5 of the GRIP protein have a short linker and a fixed domain orientation. While the binding pocket of PDZ4 is closed and the domain is not involved in the peptide interaction, the PDZ domain itself and the linker between these domains are crucial for the binding of a receptor tail to PDZ5 [22]. On the contrary, the PDZ domains 1 and 2 of PTPN13 both interact with different peptides. Previously, a binding study revealed that an allosteric effect, triggered by a domain-domain interface between PDZ1 and PDZ2, modulates the binding properties of the PDZ2 domain [21].

In this study, we have observed different binding characteristics of an APC-derived peptide ligand for the isolated PDZ2 domain in comparison to the PDZ2 domain when being part of a PTPN13 PDZ2/3 tandem construct. The reason for the different dissociation constants for PDZ2, listed in Table 1, is either the presence or, respectively, the absence of the PDZ3 domain. This is in concordance with previously described allosteric effects caused by domain-domain interaction between the PDZ domains 1 and 2 of PTPN13 [5, 21, 30]. Together with these previous results, our data now allow for the first time to generate of an overall model for the PTPN13 domain triplet PDZ1–3, that reveals the crucial role of the PDZ2 domain. The analysis of the NMR chemical shift differences between spectra of the single PDZ2-APC complex and PDZ2 as a part of the PDZ2/3-APC complex (together with the results obtained from the titration of the single PDZ3 domain with the single PDZ2 domain) suggest an interaction between the two PDZ domains 2 and 3 when PTPN13 is complexed with APC, in sharp contrast to the peptide ligand-free PDZ2/3 tandem domain of PTPN13. The numerous NMR chemical shift perturbations observed for EA and EF of PDZ2 are among the most prominent and are probably caused by the presence of the PDZ3 domain and maybe even the linker region between these two adjacent PDZ domains. These NMR chemical shift differences are indicative of a long-range allosteric effect, which is most likely caused by PDZ3 interacting with the backside of the PDZ2 domain within the APC peptide-bound PDZ2/3 tandem domain of PTPN13. These results clearly indicate that, upon titration with the APC peptide, the PDZ2 and PDZ3 domain interact with each other and the equilibrium (partly) shifts towards the PDZ2/PDZ3 complex state (Fig. 5). The HADDOCK-generated model of PDZ2/3 complex in the APC-bound state with an overall docking software score of −92.7 ± 13.9 is consistent with experimental chemical shift perturbation restraints and reveals that both canonical PDZ binding clefts are facing the solvent (Fig. 6 b, Table 2). The PDZ2/3 tandem domain interface, which contains PDZ β-strands 1 (βA) and 6 (βF) is located on the opposite site to create a buried surface area of 1215.5 ± 206.2 Å^2^ (Table 2). Despite a residual rotational freedom of the two PDZ domains within in the cluster of 8 structural models of the APC-bound PDZ2/3 tandem domain from PTPN13, the canonical PDZ binding clefts are always orientated towards the solvent (Fig. 6b). Presumably, this residual degree of rotational heterogeneity of the PDZ domains is due to the limited number of chemical shift perturbations restraints. Nonetheless, the canonical peptide binding clefts of PDZ2 and PDZ3 are distant from the PDZ2/3 tandem domain interface allowing for allosteric regulation of PTPN13 (Fig. 6b).

Furthermore, we could also observe NMR chemical shift perturbations for amino acids located in the core of the domain of PDZ2, ranging from the distal regions of the domain to the ligand-binding site in concordance with previous studies [3–5]. Based on this intra-domain allosteric modulation, the interaction with PDZ3 presumably causes crucial structural changes in the ligand binding site of PDZ2 that are located opposite to the domain interface (Figs. 5 and 6b). The NMR chemical shift perturbations are observed for the entire binding loop that consists of the binding motive, β-strand EB and α-helix DB. In conclusion, the interaction between PDZ2 and PDZ3 might induce a structural change from the interface to the binding loop of PDZ2, thereby affecting its peptide binding characteristics (Table 1). The result of this allosteric effect can be directly observed by changes of the APC dissociation constants K_D_ for PDZ2. This indicates that interaction between the PDZ-PDZ domains can indeed modify PDZ ligand binding characteristics (Table 1).

Even though the binding behaviour of PDZ domains has been intensively studied, the functional consequences are still not completely understood. Previous studies show [30] that the properties of PDZ domains must be interpreted as a whole to establish a functional model of multi-PDZ domain complexes as found in PTPN13 [21]. The biochemical and structural data on PTPN13 presented in this work clearly demonstrate two facts: firstly, PRK2 also interacts with PDZ2 thereby extending the number of known interaction partners of PDZ2 from PTPN13 [1, 8, 9]. Secondly, PDZ2 is allosterically modulated by PDZ3. The titration of PDZ2 with the APC-derived peptide reveals NMR chemical shift perturbations on the entire domain, with major changes mainly observed for hydrophobic amino acids. The appearance of NMR chemical shift differences on the entire domain suggests an allosteric network induced by the binding of the APC-derived peptide. A previous analysis of allosteric interactions in PDZ2 [3, 5, 30, 31] showed, that residues within van-der-Waals contact distance can mediate conformational changes in a PDZ domain. We propose that peptides with hydrophobic amino acids at position P_0_, like valine in the APC peptide, initiate a conformational change by a van der Waals interaction in the ligand binding site, which is propagated through a hydrophobic network within the domain. Therefore, ligands that carry a hydrophobic amino acid at position P_0_ might act as an inductor of an intra-domain structural signalling network mediated by the hydrophobic core of the PDZ domain [3–5]. This binding study of the PDZ2/3 tandem domain with the APC-derived peptide revealed that APC chooses PDZ2 over PDZ3 as a native binding partner (Table 1). It is remarkable that the binding affinity for the APC peptide towards PDZ2 in the tandem domain is slightly higher than for the single PDZ2 domain (K_D_ ~ 211 μM vs K_D_ ~ 286 μM) while the binding affinity of the APC peptide for PDZ3 is even lowered (K_D_ ~ 721 μM vs K_D_ ~ 2574 μM). The latter K_D_ value indicates that significant binding of APC to PDZ3 in the tandem domain will most likely not occur under physiological conditions.

**Fig. 7:**
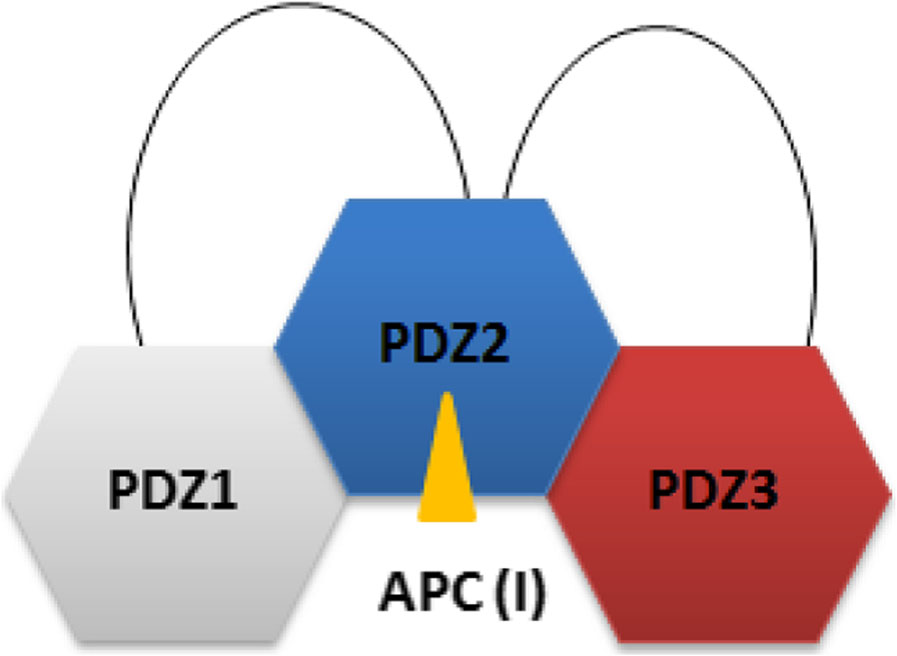
Schematic functional model of the APC-bound PDZ1/2/3 domain triplet from PTPN13. Note, the proximate domains PDZ1 and PDZ3 interact with a mutually exclusive surface patch on PDZ2 located opposite to the canonical ligand binding site of PDZ domains

Based on the results from the NMR chemical shift perturbation analysis and HADDOCK molecular docking calculations we conclude, that an allosteric effect modulates the APC-binding characteristics of PDZ2 from PTPN13 (Fig. 7). Although peptides rather than the corresponding full-length proteins have been used here, numerous studies have shown before that the last four to six carboxy-terminal amino acids are essential for ligand binding [32–34] to PDZ domains. A previous study on the binding characteristics of the PDZ domains 1 and 2 from PTPN13 clearly showed, that the single PDZ2 domain is able to bind to peptide ligands from several classes, such as I (APC) and III (RIL) [21]. In the PDZ1/2 tandem construct, however, PDZ2 binding is apparently limited to class I ligands only [21]. In our study we could now observe that, in comparison to the isolated PDZ2 domain, the binding capacity of PDZ2 in the PDZ2/3 tandem domain is slightly higher for the class I ligand APC (Table 1). The NMR line widths only slightly increase for the APC-bound form of the PDZ2/3 tandem domain (Fig. 4). This suggests an equilibrium between free and interacting PDZ domains within the PDZ2/3 tandem. Upon APC binding, this equilibrium shifts, presumably during a process of conformations selection, towards the compact form with a higher affinity of PDZ2 for, in which the state of two PDZ domains of the PDZ2/3 tandem interacting with each other is more populated (Figs. 6 and 7).

Our data on the PTPN13 PDZ2/3 tandem domain are therefore consistent with and complement findings for the PDZ1/2 tandem, as the binding affinity for the APC peptide ligand is increased in the PDZ 2/3 tandem domain in comparison to the single PDZ2 domain [21] (Table 1). Furthermore, since PDZ1 does bind to PDZ2 in the absence of ligand, PDZ3 apparently has to compete with PDZ1 in the full-length molecule or at least in the PDZ1/2/3 domain triplet [21] (Fig. 7). Obviously, PDZ2 is regulated by the adjacent PDZ domains 1 and 3 in the triplet PDZ123 domain cassette and is thus pivotal as most known binding partners of PTPN13 interact with the PDZ2 domain that also includes PRK2 [1]. In the tandem PDZ2/3 domain of PTPN13, PDZ2 apparently exhibits an increased affinity for class I ligands, such as APC (Table 1). In full-length PTPN13 however, this binding behaviour might be even further modulated by PDZ1.

This modulation of binding affinities of PTPN13 for its ligands might also lead to the formation of supramolecular protein complexes through which PTPN13 exhibits its well established scaffolding functions in several cellular processes such as cytokinesis and actin-cytoskeletal rearrangement [1]. This might also explain as to why the observed in vitro affinities of PTPN13 for APC and PRK2 are low in comparison to known examples for PDZ-ligand interactions [1, 35–37]. Interestingly, our finding that the C-terminus of PRK2 can, in principle, bind to two adjacent PDZ domains in mouse PTPN13 could be of physiological relevance. It has been demonstrated that PRK2 can form dimers. The amino-terminus of PRK2 can bind to the kinase domain of PRK2 in trans leading to an inactive dimer [38]. The weak binding affinity we observed for binding to the individual PDZ domains might help to discriminate between binding to monomeric or dimeric PRK2, as only the latter would bind with higher affinity given its potential simultaneous binding to two PDZ domains. Along this line it has been demonstrated that the PDZ4 domain of PTPN13 binds the RhoGAP protein PARG1, which in principle could be a negative regulator of Rho-dependent PRK2 [39]. It is conceivable that the PDZ2–4 domains form a module that mechanistically contributes to the spatial inactivation of PRK2. This could be important for example for the coordinated inactivation of PRK2 in vivo during regulation of cytokinesis, a process PTPN13 and PRK2 have both been implicated in [40, 41].

## Conclusions

Taken together, our study suggests that the PDZ2 of PTPN13 plays a central role in the triplet cassette PDZ123 and that PDZ2 is regulated by the adjacent PDZ domains 1 and 3 (Fig. 7). This is in agreement with a previously published study [21] on PDZ1/2. Based on NMR chemical shift perturbation experiments and a molecular HADDOCK model of the PDZ2/3 tandem domain from PTPN13, the proximate domains PDZ1 and PDZ3 interact with a mutually exclusive surface patch on PDZ2 located opposite to the canonical ligand binding site of PDZ domains, which mainly consists of the two β-strands EA and EF (Figs. 5 and 6a, b). In conclusion, an extended model of PDZ1–3 could be established that stresses the central role of PDZ2 in the PDZ1/2/3 domain triplet within APC-bound PTPN13 (Fig. 7). Despite the linker sequences located between them, the first three PDZ domains of PTPN13 might hence modulate their affinity for physiological targets, at least for the PDZ2/APC interaction.

## Methods

### Molecular biology

Bacterial expression plasmids pGEX-2 T-PDZ2 (PTPN13 residues 1347–1450), pGEX-2 T-PDZ3 (PTPN13 residues 1474–1580), and pGEX-2 T-PDZ2/3 (PTPN13 residues 1347–1580) were constructed by sub-cloning of PCR-generated PTPN13 cDNA fragments in-frame into appropriate pGEX-2 T vectors.

### Isotopic enrichment of proteins

As previously published, isotope-labeled proteins were prepared by growing *E. coli* BL21 (DE3) cells in isotope-enriched minimal media using ^13^C glucose and/or ^15^N ammonium chloride as carbon and nitrogen sources [25, 42–47].

### Expression and purification of PDZ domains from PTPN13

GST-fusion proteins were expressed in *E. coli* BL21(DE3) under ampicillin and chloramphenicol selection. In order to generate ^15^N isotopically-enriched proteins, cells were grown in minimal media [25]. Proteins with a natural isotope distribution were produced at 37 °C in Luria broth media [48, 49]. In either case, cells were grown to an optical density of approx. 0.7 (measured at 595 nm) and protein expression was induced by 0.75 mM IPTG. Then, the cells were grown for an additional period of 16 h. After increasing the IPTG concentration to 1 mM, the cells were incubated for an additional period of 2 h. The cells were harvested and resuspended in phosphate buffered saline (PBS) at pH 7.4 and EDTA-free protease inhibitors (Roche) were added before cells were lysed in a micro fluidiser (*Microfluidics Corporation*. Cell debris was removed by centrifugation at 10,200 g and at 4 °C for 45 min. Afterwards, the supernatant was incubated with Glutathione Sepharose 4B beads (GE Healthcare) at 20 °C for 60 min. The GST-tag was subsequently cleaved off through incubation with Thrombin protease (GE Healthcare) for 12 h at 4 °C. Then, the protein was eluted from the Glutathione Sepharose beads with phosphate buffered saline (PBS) at 20 °C. Protein containing fractions were pooled and concentrated in a Millipore concentrator with molecular weight cut-offs of 5000–10,000 Da, frozen in liquid nitrogen, and finally stored at − 80 °C until further use. The integrity of purified proteins was checked by SDS-PAGE and mass spectrometry (data not shown).

### NMR spectroscopy

NMR spectra were acquired at 298 K on Bruker DRX 600 and AVANCE III HD 700 spectrometers. Typically, NMR samples contained 0.1–0.5 mM uniformly ^13^C and/or ^15^N-enriched protein in PBS buffer at pH 7.4 including 10% D_2_O [42–44]. All data were processed with either NMRPipe [50] and analyzed with NMR View [51] or TopSpin (www.bruker.com). The backbone assignments were obtained from the BioMagResBank accession numbers 15,199 (PDZ2/3) [25] and 5131 (PDZ2) [25–27, 42, 44, 46]. The interaction between PDZ2 and PDZ3 was analyzed upon titrating PDZ3 to isotopically ^15^N-enriched PDZ2 at a molar ratio of 1:1 at 298 K by recording 2D ^1^H-^15^N-HSQC NMR spectra. In this study, the numbering scheme of the assigned chemical shifts for PDZ2 (5131) and PDZ23 (15199) has been shifted by one residue so that, for example, T30 according to the BMRB entry 5131 now equals T31. Amino-terminally acetylated dodecapeptides were commercially obtained from JPT Peptide Technologies (Germany). The peptide sequences KRHSGSYLVTSV (M = 1332.69 g/mol) and MFRDFDYIADWC (M = 1580.64 g/mol) correspond to the last 12 C-terminal residues of APC and PRK2, respectively. APC and PRK2 peptides were titrated with PDZ2, PDZ3, and PDZ2/3 domains that were isotopically enriched with ^15^N. The experiments were carried out at protein-to-ligand ratios of 1:0, 1:1, 1:3, 1:5, 1:10, 1:20, and 1:30 at 298 K and pH 7.4. For all binding studies that included a PDZ3 domain, Dithiothreitol (DTT) was added to the sample before carrying out the titration in order to avoid the oxidation of cysteines. The differences in backbone ^1^H and ^15^N NMR chemical shifts were monitored via 2D ^1^H-^15^N-HSQC NMR spectra. The weighted ^1^H_N_ and ^15^N chemical shift differences were calculated according to the following equation [45, 47]:

$$ \varDelta {\delta}_{obs}\kern0.5em =\kern0.5em \sqrt{{\left(\varDelta {\delta}_{1_{H_N}}\right)}^2\kern0.5em +\kern0.5em {\left(\frac{\varDelta {\delta}_{15_N}}{5}\right)}^2} $$

Averaged dissociation constants for the different complexes were calculated from least-squares fitting of the NMR chemical shift perturbations observed for several amino acids as a function of ligand concentration. NMR chemical shift perturbation data were analysed with the ORIGIN software package (www.originlab.com) as previously published [12, 52]. The error for K_D_ values was calculated from the individual fit of several significant chemical shift perturbations found for the amide resonances located in and/or next to the canonical PDZ binding cleft. For the PDZ2/APC titration amide resonances of G23, K50, A52, L73, Q80, and A81 of were used, for the PDZ2/PRK2 titration amide resonances of T21, G23, T30, Y43, G51, A52, S55, and D56 were used, for the ‘PDZ2 in PDZ2/3’/APC titration amide resonances of L19, D57, and Q81 were used, and, finally, for the ‘PDZ3 in PDZ2/3’/APC amide resonances of G159, F162, S163, K184, L223, and G225 were used.

### Molecular dynamics docking

The High Ambiguity Driven protein-protein DOCKing (HADDOCK) software package was used to generate a model of the APC-bound PDZ2/3 tandem from PTN13 based on the differential NMR chemical shift perturbations outside the canonical binding cleft of PDZ domains between PDZ2/APC and ‘PDZ2 in PDZ2/3’/APC titration [28]. For PDZ2 [27], T14, F15, E16, E18, N70, H94, and L95 were selected as active ambiguous interaction restraints (AIRs). These surface-exposed residues are located opposite to the canonical PDZ binding cleft. T14, F15, E16, and E18 are located on β-strand EA and H94, L95 on β-strand EF. For a homology model of PDZ3, E17, V18, K19, L20, S98, and L100 were set as active AIRs. These residues are also positioned on β-strand EA (E17, V18, K19, and L20) and β-strand EF (S98 and L100). Passive AIRs were automatically defined by HADDOCK [28]. The molecular dynamics-based docking is driven by the experimental restraints, i. e. nmr chemical shift perturbations extracted from the PTPN13/APC titrations experiments in this case [28]. In HADDOCK, the entire CHARMM force fields-based docking procedure includes rigid-body energy minimization and semi-flexible refinement using torsion angle molecular dynamics, followed by refinement in explicit water [28].

## Data Availability

The backbone ^1^H, ^13^C, and ^15^N chemical shift assignments of the PDZ2/3 tandem domain of PTPN13 can be found under the BioMagResBank accession number 15199.

## Abbreviations

APC: Tumour suppressor protein adenomatous polyposis coli
FERM: Four point one, Ezrin, Radixin, Moesin
GRIP: Glutamate receptor interacting protein
HADDOCK: High ambiguity driven protein-protein docking
HSQC: Heteronuclear single quantum coherence
KIND: Kinase non-catalytic C-lobe domain
LEF/TCF: Lymphocyte enhancer binding factor/T-cell factor
NMR: Nuclear magnetic resonance
PDZ: PSD95, Disc-large, occludens-1
PRK2: Protein kinase C-related kinase-2
PTP: Protein tyrosine phosphatase PTP-Basophil/Basophil-like
PTPN13: Tyrosine-protein phosphatase non-receptor type 13

## References

[1] Erdmann KS (2003). The protein tyrosine phosphatase PTP-basophil/basophil-like. Interacting proteins and molecular functions. Eur J Biochem.

[2] Feng W, Zhang M (2009). Organization and dynamics of PDZ-domain-related supramodules in the postsynaptic density. Nat Rev Neurosci.

[3] Kong Y, Karplus M (2009). Signaling pathways of PDZ2 domain: a molecular dynamics interaction correlation analysis. Proteins.

[4] Fuentes EJ, Der CJ, Lee AL (2004). Ligand-dependent dynamics and intramolecular signaling in a PDZ domain. J Mol Biol.

[5] Lockless SW, Ranganathan R (1999). Evolutionarily conserved pathways of energetic connectivity in protein families. Science.

[6] Doyle DA, Lee A, Lewis J, Kim E, Sheng M, MacKinnon R (1996). Crystal structures of a complexed and peptide-free membrane protein-binding domain: molecular basis of peptide recognition by PDZ. Cell.

[7] Morais Cabral JH, Petosa C, Sutcliffe MJ, Raza S, Byron O, Poy F, Marfatia SM, Chishti AH, Liddington RC (1996). Crystal structure of a PDZ domain. Nature.

[8] Erdmann KS, Kuhlmann J, Lessmann V, Herrmann L, Eulenburg V, Muller O, Heumann R (2000). The adenomatous polyposis coli-protein (APC) interacts with the protein tyrosine phosphatase PTPN13 via an alternatively spliced PDZ domain. Oncogene.

[9] Gross C, Heumann R, Erdmann KS (2001). The protein kinase C-related kinase PRK2 interacts with the protein tyrosine phosphatase PTPN13 via a novel PDZ domain binding motif. FEBS Lett.

[10] Long JF, Tochio H, Wang P, Fan JS, Sala C, Niethammer M, Sheng M, Zhang M (2003). Supramodular structure and synergistic target binding of the N-terminal tandem PDZ domains of PSD-95. J Mol Biol.

[11] Vaccaro P, Dente L (2002). PDZ domains: troubles in classification. FEBS Lett.

[12] Kock G, Dicks M, Yip KT, Kohl B, Pütz SH, Erdmann KS, Stoll R (2018). Molecular basis of class III ligand recognition by PDZ3 in murine protein tyrosine phosphatase PTPN13. J Mol Biol.

[13] Stiffler MA, Chen JR, Grantcharova VP, Lei Y, Fuchs D, Allen JE, Zaslavskaia LA, MacBeath G (2007). PDZ domain binding selectivity is optimized across the mouse proteome. Science.

[14] Tonikian R, Zhang Y, Sazinsky SL, Currell B, Yeh JH, Reva B, Held HA, Appleton BA, Evangelista M, Wu Y, Xin X, Chan AC, Seshagiri S, Lasky LA, Sander C, Boone C, Bader GD, Sidhu SS (2008). A specificity map for the PDZ domain family. PLoS Biol.

[15] Vaccaro P, Brannetti B, Montecchi-Palazzi L, Philipp S, Helmer Citterich M, Cesareni G, Dente L (2001). Distinct binding specificity of the multiple PDZ domains of INADL, a human protein with homology to INAD from Drosophila melanogaster. J Biol Chem.

[16] Behrens J, von Kries JP, Kuhl M, Bruhn L, Wedlich D, Grosschedl R, Birchmeier W (1996). Functional interaction of beta-catenin with the transcription factor LEF-1. Nature.

[17] Tan C, Costello P, Sanghera J, Dominguez D, Baulida J, de Herreros AG, Dedhar S (2001). Inhibition of integrin linked kinase (ILK) suppresses beta-catenin-Lef/Tcf-dependent transcription and expression of the E-cadherin repressor, snail, in APC−/− human colon carcinoma cells. Oncogene.

[18] Hulsken J, Behrens J, Birchmeier W (1994). Tumor-suppressor gene products in cell contacts: the cadherin-APC-armadillo connection. Curr Opin Cell Biol.

[19] Flynn P, Mellor H, Casamassima A, Parker PJ (2000). Rho GTPase control of protein kinase C-related protein kinase activation by 3-phosphoinositide-dependent protein kinase. J Biol Chem.

[20] Vincent S, Settleman J (1997). The PRK2 kinase is a potential effector target of both rho and Rac GTPases and regulates actin cytoskeletal organization. Mol Cell Biol.

[21] van den Berk LC, Landi E, Walma T, Vuister GW, Dente L, Hendriks WJAJ (2007). An allosteric intramolecular PDZ-PDZ interaction modulates PTP-BL PDZ2 binding specificity. Biochemistry.

[22] Feng W, Shi Y, Li M, Zhang M (2003). Tandem PDZ repeats in glutamate receptor-interacting proteins have a novel mode of PDZ domain-mediated target binding. Nat Struct Biol.

[23] Long JF, Feng W, Wang R, Chan LN, Ip FC, Xia J, Ip NY, Zhang M (2005). Autoinhibition of X11/mint scaffold proteins revealed by the closed conformation of the PDZ tandem. Nat Struct Mol Biol.

[24] van den Berk LC, Landi E, Harmsen E, Dente L, Hendriks WJ (2005). Redox-regulated affinity of the third PDZ domain in the phosphotyrosine phosphatase PTPN13 for cysteine-containing target peptides. FEBS J.

[25] Fetzer CP, Sauvageau J, Kock G, Berghaus C, Bangert JA, Dicks M, Heumann R, Erdmann KS, Stoll R (2007). Sequence-specific (1)H, (13)C, and (15)N backbone assignment of the 28 kDa PDZ2/PDZ3 tandem domain of the protein tyrosine phosphatase PTPN13. Biomol NMR Assign.

[26] Walma T, Aelen J, Nabuurs SB, Oostendorp M, van den Berk L, Hendriks W, Vuister GW (2004). A closed binding pocket and global destabilization modify the binding properties of an alternatively spliced form of the second PDZ domain of PTPN13. Structure.

[27] Walma T, Spronk CA, Tessari M, Aelen J, Schepens J, Hendriks W, Vuister GW (2002). Structure, dynamics and binding characteristics of the second PDZ domain of PTPN13. J Mol Biol.

[28] Dominguez C, Boelens R, Bonvin AM (2003). HADDOCK: a protein-protein docking approach based on biochemical or biophysical information. J Am Chem Soc.

[29] Long J, Wei Z, Feng W, Yu C, Zhao YX, Zhang M (2008). Supramodular nature of GRIP1 revealed by the structure of its PDZ12 tandem in complex with the carboxyl tail of Fras1. J Mol Biol.

[30] Gianni S, Walma T, Arcovito A, Calosci N, Bellelli A, Engstrom A, Travaglini-Allocatelli C, Brunori M, Jemth P, Vuister GW (2006). Demonstration of long-range interactions in a PDZ domain by NMR, kinetics, and protein engineering. Structure.

[31] Ota N, Agard DA (2005). Intramolecular signaling pathways revealed by modeling anisotropic thermal diffusion. J Mol Biol.

[32] Kornau HC, Seeburg PH (1997). Partner selection by PDZ domains. Nat Biotechnol.

[33] Ponting CP, Phillips C, Davies KE, Blake DJ (1997). PDZ domains: targeting signalling molecules to sub-membranous sites. Bioessays.

[34] Fanning AS, Anderson JM (1996). Protein-protein interactions: PDZ domain networks. Curr Biol.

[35] Saro D, Li T, Rupasinghe C, Paredes A, Caspers N, Spaller MR (2007). A thermodynamic ligand binding study of the third PDZ domain (PDZ3) from the mammalian neuronal protein PSD-95. Biochemistry.

[36] Milev S, Bjelic S, Georgiev O, Jelesarov I (2007). Energetics of peptide recognition by the second PDZ domain of human protein tyrosine phosphatase 1E. Biochemistry.

[37] Tonikian R, Zhang Y, Sazinsky SL, Curell B, Yeh J-H, Reva B, Held HA, Appleton BA, Evangelista M, Wu Y, Xin X, Chan AC, Seshargiri S, Laskz LA, Sander C, Boone C, Bader GB, Sidhu SS (2008). A specificity map for the PDZ domain family. PLoS Biol.

[38] Bauer AF, Sonzogni S, Meyer L, Zeuzem S, Piiper A, Biondi RM, Neimanis S (2012). Regulation of protein kinase C-related protein kinase 2 (PRK2) by an intermolecular PRK2-PRK2 interaction mediated by its N-terminal domain. J Biol Chem.

[39] Saras J, Franzen P, Aspenstrom P, Hellmann U, Gonez LJ, Heldin C-H (1997). A novel GTPase-activating protein for rho interacts with a PDZ domain of the protein-tyrosine phosphatase PTPL1. J Biol Chem.

[40] Schmidt A, Durgan J, Magalhaes A, Hall A (2007). Rho GTPases regulate PRK2/PKN2 to control entry into mitosis and exit from cytokinesis. EMBO J.

[41] Herrmann L, Dittmar T, Erdmann KS (2003). The protein tyrosine phosphatase PTP-BL associates with the Midbody and is involved in the regulation of cytokinesis. Mol Biol Cell.

[42] Berghaus C, Schwarten M, Heumann R, Stoll R (2007). Sequence-specific 1H, 13C, and 15N backbone assignment of the GTPase rRheb in its GDP-bound form. Biomol NMR Assign.

[43] Nowaczyk M, Berghaus C, Stoll R, Rogner M (2004). Preliminary structural characterisation of the 33 kDa protein (PsbO) in solution studied by site-directed mutagenesis and NMR spectroscopy. Phys Chem Chem Phys.

[44] Schwarten M, Berghaus C, Heumann R, Stoll R (2007). Sequence-specific 1H, 13C, and 15N backbone assignment of the activated 21 kDa GTPase rRheb. Biomol NMR Assign.

[45] Stoll R, Lee BM, Debler EW, Laity JH, Wilson IA, Dyson HJ, Wright PE (2007). Structure of the Wilms tumor suppressor protein zinc finger domain bound to DNA. J Mol Biol.

[46] Stoll R, Renner C, Ambrosius D, Golob M, Voelter W, Buettner R, Bosserhoff AK, Holak TA (2000). Sequence-specific 1H, 13C, and 15N assignment of the human melanoma inhibitory activity (MIA) protein. J Biomol NMR.

[47] Rehmann H, Bruning M, Berghaus C, Schwarten M, Kohler K, Stocker H, Stoll R, Zwartkruis FJ, Wittinghofer A (2008). Biochemical characterisation of TCTP questions its function as a guanine nucleotide exchange factor for Rheb. FEBS Lett.

[48] Bertani G (1951). Studies on lysogenesis. I. the mode of phage liberation by lysogenic Escherichia coli. J Bacteriol.

[49] Bertani G (2004). Lysogeny at mid-twentieth century: P1, P2, and other experimental systems. J Bacteriol.

[50] Delaglio F, Grzesiek S, Vuister GW, Zhu G, Pfeifer J, Bax A (1995). NMRPipe: a multidimensional spectral processing system based on UNIX pipes. J Biomol NMR.

[51] Johnson BA, Blevins RA (1994). Nmr view - a computer-program for the visualization and analysis of Nmr data. J Biomol NMR.

[52] Williamson MP (2013). Using chemical shift perturbation to characterise ligand binding. Prog Nucl Magn Reson Spectrosc.

